# Book Notices

**Published:** 1867-02

**Authors:** 


### Book Notices.

Proceedings of the American Pharmaceutical Association at the Fourteenth Annual Meeting, held in Detroit, Mich., August, 1866. Also the Constitution and Roll of Members, Philadelphia: MERRIHEW & SON, Printers. 1866.

This is a very neatly printed volume of 316 pages, containing the reports, essays, and proceedings presented at the last Annual Meeting. We should think it a work of great value to every intelligent druggist and pharmaceutist, and several of the reports and papers are equally interesting and valuable to the physician. We think copies can be obtained by applying to E. H. SARGENT, Druggist, corner of Randolph and State Streets, Chicago.

Diphtheria; A Prize Essay. By E. S. GAILLARD, M.D., Richmond, Va. Reprinted from the *Richmond Medical Journal.*

This is a monograph of 114 pages. Of the many essays written upon this subject during the last ten years, this is certainly one of the best. The author devotes a large part of his work to the elucidation of the pathology of the disease. He regards the disease as a general one, involving a morbid condition of the whole mass of blood, and the development of certain local inflammations of an asthenic character. Of necessity, treatment founded on such views of the pathology, must be corrective and tonic. The correctness of this has been shown by observation at the bedside.

On the Relations which Electricity sustains to the Causes of

Disease. By S. LITTELL, M.D., Emeritus Surgeon of Will's Hospital, Philadelphia. From the *Transactions of the American Medical Association.*

This is a report of 54 pages, originally presented to the American Medical Association, in Boston, 1865, but not printed in the *Transactions* until the following year. It is an exceedingly interesting and important paper. We have only space for the following summary of doctrines which are appended to the report:—

"1st. That heat and electricity are identical, as the one can be converted into the other.

2d. That a large volume of electricity surrounds every primary constituent of matter, especially that form of matter which constitutes the gaseous bodies.

3d. That animal heat is supported by the electricity liberated from the primary constituents of matter during the processes of respiration, digestion, and assimilation.

4th. That electricity is evolved during these processes on the same principle as that which is evolved during the action of a galvanic arrangement.

5th. That electricity and nervous power are analogous, if not identical, as the action of one can be successfully substituted for the other.

6th. That the majority of diseases are caused either by the sudden obstruction, or slow abduction of electricity from the body.

7th. That a low state of electric tension on the surface of the earth, produced either by the operation of evaporation, or some occult movement in the great internal currents of the earth is the remote cause of epidemic and pestilential diseases.

8th. That occasional and ordinary diseases are produced by the sudden abstraction or slow subduction of electricity from the body, or its sudden elimination during the vital processes.

9th. That since electricity is so essential to the integrity of the vital operations, it is indispensable that measures be taken to produce its evolution, and to prevent over-radiation.

10th. That electricity is the source of vitality in vegetable life.

11th. That electricity is attracted by the fibres of the roots of plants; and by the instrumentality of the electric fluid does the plant attract its constituents from the soil.

12th. That vegetables of rapid growth require a large supply of electricity to secure their perfection and completion; and the potato is a plant of this kind.

13th. That the disease in the potato was produced by the want of nutrition.

14th. That the want of nutrition arose from defective electric agency.

15th. That the cause of the deficiency of this agency was those abstracting influences which produced low tenison of electricity."

---

Reduction of Inverted Uteri, by a New Method. By THOMAS ADDIS EMMET, M.D., Surgeon in charge of New York State Women's Hospital.

This is a very neatly printed pamphlet of 15 pages, detailing two cases of reduction of inverted uteri by upward pressure, simultaneous with efforts to dilate the constricting part around the part of the organ last inverted. The first case had existed seven months, and the time consumed in effecting a reduction was *three hours and fifty-five minutes*, the patient being fully under the anæsthetic influence of ether all the time. The second case was one of eight months duration, and was reduced in *one hour and twenty minutes*. These cases, as related by Dr. EMMET, are of great interest and value, and should be read in detail by the profession.

---

Clinical Observations on Functional Nervous Disorders. By C. HANDFIELD JONES, M.B., Cantab.; F.R.C.P., London; F.R.S.; Physician to St. Mary's Hospital. Philadelphia: HENRY C. LEA. 1867. pp. 348.

For sale by W. B. KEEN & Co., 148 Lake Street.

Infantile Paralysis, and the Attendant Deformities. By CHAS. FAYETTE TAYLOR, M.D., etc., etc., etc. Philadelphia: J. B. LIPPINCOTT & Co. 1867. pp. 119. Duodecimo.

For sale by S. C. GRIGGS & Co., 41 Lake Street.

Notes on Epidemics. For the Use of the Public. By FRANCIS EDMUND ANSTIE, M.D., F.R.C.P., Senior-Assistant Physician to the Westminster Hospital. First American Edition. Philadelphia: J. B. LIPPINCOTT & Co. 1866. pp. 95. Duodecimo.

For sale by S. C. GRIGGS & Co., 41 Lake Street.

The Common Nature of Epidemics, and their Relations to Climate and Civilization. Also Remarks on Contagion and Quarantine. From Writings and Official Reports. By SOUTHWOOD SMITH, M.D., Physician to the London Fever Hospital, etc., etc., etc. Edited by T. BAKER, Esq. Philadelphia: J. B. LIPPINCOTT & Co. 1866. pp. 130.

For sale by S. C. GRIGGS & Co., 41 Lake St. Price $1.50.

Lessons upon the Diagnosis and Treatment of Surgical Diseases, Delivered in the month of August, 1865. By Professor VELPEAU, Membre de l'Institute et de l'Académie de Médecine. Collected and edited by A. REGNARD, Interne des Hôpitaux. Reviewed by the Professor. Translated by W. C. B. FIFIELD, M.D. Boston: JAMES CAMPBELL. 1866. Duodecimo. pp. 103. Price $1.00

